# Supplementary material for: Moving Away from 12:12; the Effect of Different Photoperiods on Biomass Yield and Cannabinoids in Medicinal Cannabis
Source: Plants (Basel). 2023 Feb 27;12(5):1061. doi: 10.3390/plants12051061 (PMC10004775; doi:10.3390/plants12051061)
Supplement: Supplementary file 1 [file plants-12-01061-s001.zip › plants-2189056-supplementary.pdf]

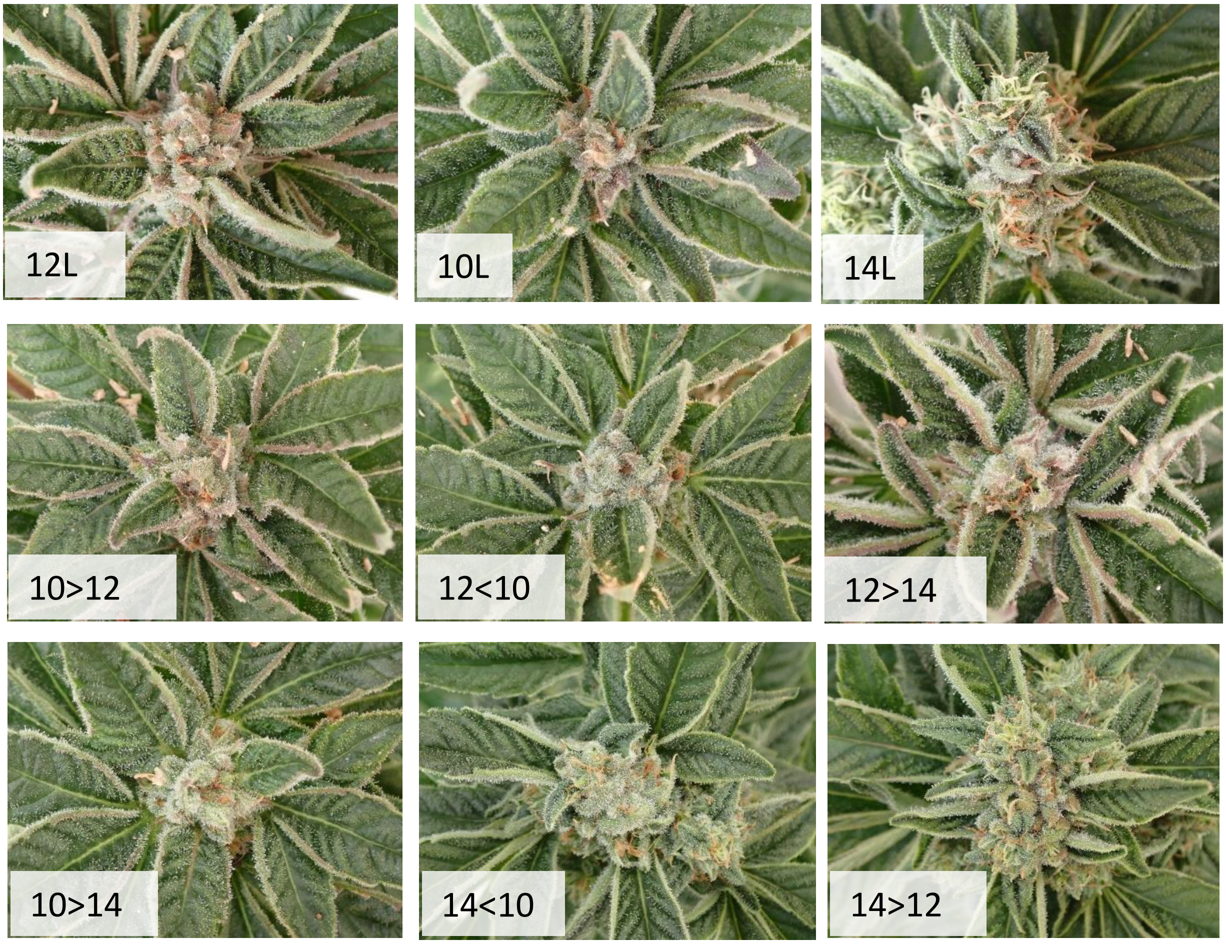


Figure S1: Photographs of the top of the main flowering stem of Cannatonic taken at DAC 67 from all nine treatments

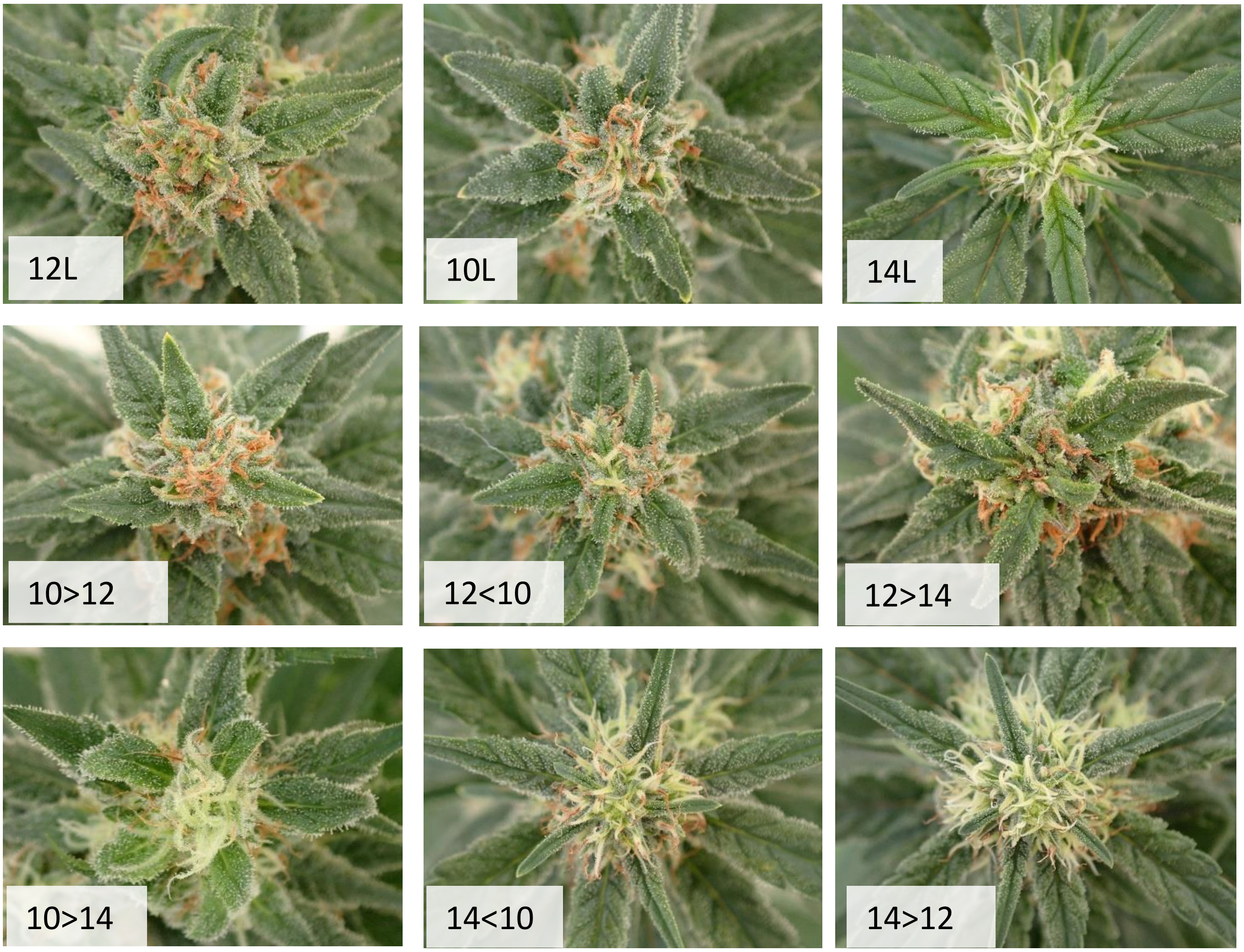


Figure S2: Photographs of the top of the main flowering stem of Northern Lights taken at DAC 67 from all nine treatments

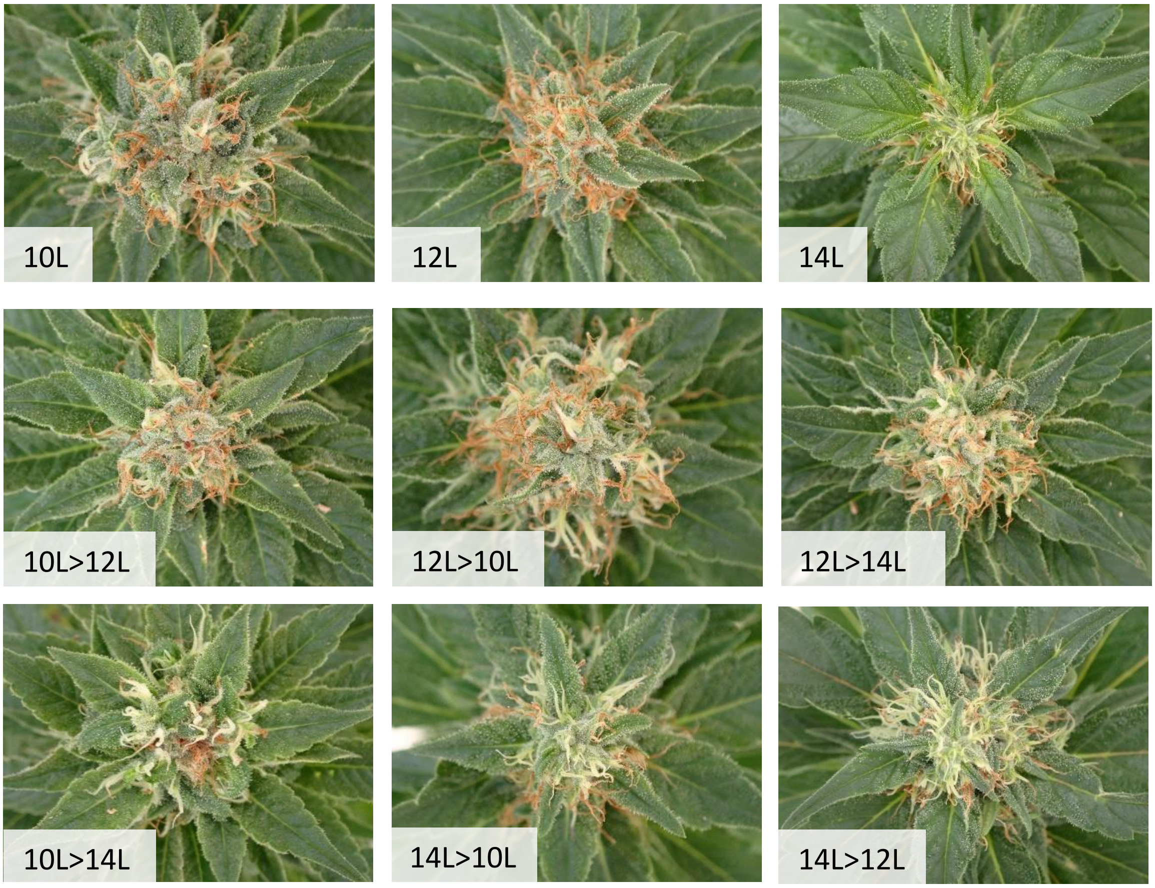


Figure S3: Photographs of the top of the main flowering stem of Hindu Kush taken at DAC 67 from all nine treatments
